# A Case Report of Migraine With Hypoplasia of the Internal Carotid Artery

**DOI:** 10.7759/cureus.45497

**Published:** 2023-09-18

**Authors:** Rafa H Alsharif, Raghad M Alhussain, Shahad A Alotaibi, Jawaher I Alraihan, Ahlam Alharbi

**Affiliations:** 1 General Practice, Taibah University, Medina, SAU; 2 General Practice, Khamis Mushait General Hospital, Aseer, SAU; 3 College of Medicine, Sulaiman Al Rajhi University, Al Bukayriyah, SAU; 4 General Practice, King Faisal University, Hofuf, SAU; 5 Family Medicine, Primary Health Care Center, Riyadh, SAU

**Keywords:** case report, magnetic resonance angiography, magnetic resonance imaging, hypoplasia, internal carotid artery, migraine

## Abstract

Migraine, a widespread and incapacitating neurological disorder, affects numerous individuals worldwide, causing severe headaches and impairing their quality of life. The interplay of genetic, environmental, and neurovascular factors underlies the pathophysiology of migraine. This report highlights the case of a 25-year-old woman with recurrent, severe headaches, predominantly in the right frontal and temporal regions. She was diagnosed with migraine with aura, a diagnosis supported by her family history. No previous history of seizures was reported. A comprehensive work-up, including neuroimaging, revealed left internal carotid artery hypoplasia with compensatory collateral circulation. The coexistence of severe migraines and left internal carotid artery hypoplasia underscores the complex interrelationship between cerebrovascular anomalies and neurological symptoms. The rarity of this vascular variation emphasizes the need for attentive clinical evaluation and consideration of anatomical deviations in migraine patients. As medical knowledge progresses, further research is essential to unravel the mechanisms connecting vascular anomalies and neurological disorders, ultimately leading to personalized interventions for improved patient outcomes.

## Introduction

Migraine, a common and debilitating neurological disorder, is characterized by recurrent episodes of severe headaches accompanied by a constellation of symptoms that can significantly impact a patient's quality of life [1]. It is estimated that over one billion individuals worldwide are affected by migraines, making it one of the leading causes of years lived with a disability. Migraine presents as a complex interplay of genetic, environmental, and neurovascular factors, and its pathophysiology involves intricate neuronal and vascular mechanisms [2].

The pathophysiological basis of migraine encompasses cortical spreading depression, altered serotonin neurotransmission, trigeminovascular activation, and vascular changes. Moreover, genetic predisposition and environmental triggers further contribute to the variable clinical presentation and frequency of attacks [2,3]. In this case report, we present a clinical account of a 25-year-old woman diagnosed with migraines, who was found to have an intriguing finding of hypoplasia of the left internal carotid artery as an incidental radiological observation. This case underscores the importance of potential coincidental anatomical variations that can be encountered in patients with migraines.

## Case presentation

A 25-year-old woman with a history of recurrent severe headache episodes presented to the neurology outpatient clinic. The patient reported experiencing throbbing headaches localized primarily to the right frontal and temporal regions. The headaches were characterized by their sudden onset, lasting for approximately four to 72 hours, and were frequently accompanied by photophobia, phonophobia, and nausea. The intensity of the pain often forced her to seek a quiet and dark room. She reported a family history of migraine, with her mother and sister experiencing similar symptoms.

Upon physical examination, the patient appeared in no acute distress. Her vital signs were stable, and a general physical examination did not reveal any abnormalities. Neurological examination findings were within normal limits, including a normal cranial nerve assessment. However, palpation of the temporal arteries did not elicit any tenderness, and there were no signs of cranial nerve deficits. The rest of the neurological examination, including motor, sensory, coordination, and reflex assessments, yielded unremarkable results.

The differential diagnosis was carefully considered. Given the patient's clinical presentation, family history, and imaging findings, the primary diagnosis of migraine with aura was favored. However, it was crucial to differentiate this from other conditions such as tension-type headache, cluster headache, and secondary headaches due to underlying structural abnormalities. The absence of focal neurological deficits, sudden severe pain, or other alarming signs made intracranial bleeding less likely.

**Figure 1:**
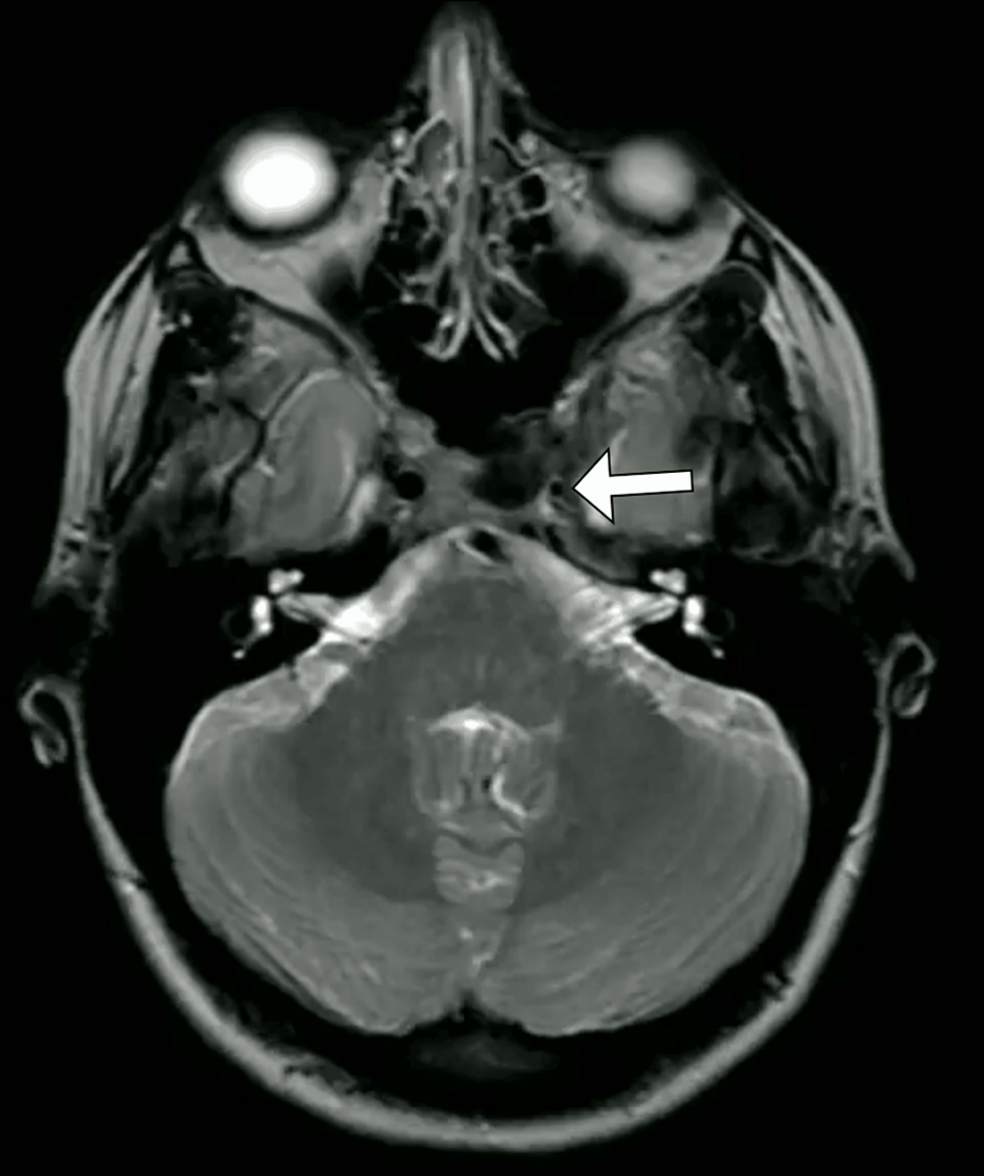
T2-weighted brain magnetic resonance image highlighting the constriction of the left internal carotid artery (arrow).

**Figure 2:**
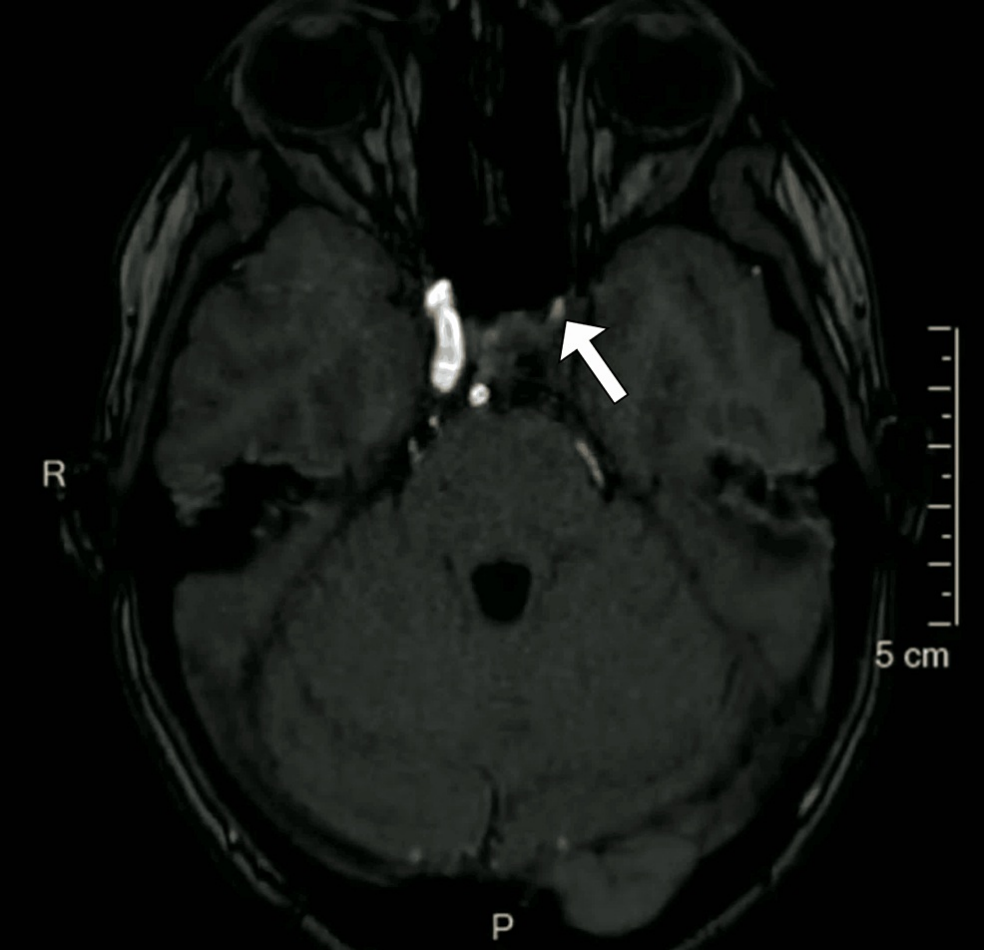
Magnetic resonance cerebral angiography image highlighting the diminished diameter of the left internal carotid artery (arrow).

In light of her recurrent and characteristic headache symptoms, a comprehensive work-up was initiated. Initial laboratory investigations, including complete blood count, electrolytes, renal and liver function tests, and C-reactive protein levels, were all within normal ranges. The coagulation profile was also normal. To further evaluate her headaches and rule out secondary causes, a magnetic resonance imaging of the brain was performed. The magnetic resonance imaging revealed hypoplasia of the left internal carotid artery with compensatory prominence of the collateral internal carotid artery (Figure 1). Magnetic resonance angiography confirmed the presence of the left internal carotid artery hypoplasia and demonstrated adequate collateral circulation through the circle of Willis (Figure 2). No other structural abnormalities, tumors, or vascular malformations were identified in the imaging studies.

The patient was educated about the nature of her condition and the role of lifestyle modifications, stress management, and trigger avoidance in migraine management. She was also provided with a tailored pharmacological regimen involving acute attack management using non-steroidal anti-inflammatory drugs and triptans, as well as prophylactic therapy with beta-blockers to reduce the frequency and severity of migraine attacks. Regular follow-up appointments were scheduled to monitor her response to treatment and adjust the management plan if necessary.

Throughout her hospital course, the patient's symptoms gradually improved with the prescribed treatment regimen. She reported a reduction in the frequency and intensity of her headache episodes. The importance of compliance with the treatment plan and maintaining a headache diary to track triggers and responses to therapy was emphasized during her follow-up visits.

## Discussion

The incidental discovery of hypoplasia of the left internal carotid artery in the context of this migraine case presents an intriguing confluence of vascular variation and neurological symptoms. The internal carotid artery rarely exhibits developmental mutations. Recognition of such aberrations becomes paramount, especially when considering potential endovascular interventions for cerebrovascular events necessitating revascularization [4,5]. While cases of the hypoplastic internal carotid artery are occasionally encountered due to collateral circulation compensation into the anterior and middle cerebral arteries, clinical symptoms are typically absent. However, in some instances, patients may manifest symptoms such as headaches, transient ischemic attacks, cranial nerve deficits, and Horner's syndrome [6].

The vascular consequences of hypoplastic internal carotid artery encompass a spectrum of intracranial events [5]. Aneurysm formation within the posterior circulation, driven by hemodynamic stress through collateral circulation, is a prevalent outcome. Acute ischemic attacks are another consequence, particularly among individuals with an incomplete circle of Willis and inadequate collateral circulation. Cerebral artery thrombosis has also been documented, further emphasizing the potential impact of hypoplasia on cerebral circulation and neurological function [6].

Clinically, congenital hypoplasia of the internal carotid artery frequently remains asymptomatic due to effective compensation by collateral circulation. However, rare instances may result in headaches, seizures, transient ischemic attacks, and even subarachnoid hemorrhage. Complications such as trigeminal neuralgia, oculomotor paralysis, visual field defects, and vision loss can stem from basilar artery and anterior communicating artery dilation or aneurysm formation [4]. Intriguingly, while the general population's average prevalence of cerebrovascular aneurysms lies between 2% and 4%, a higher incidence of 27.8% has been noted in individuals with congenital hypoplasia of the internal carotid artery [7].

In terms of epidemiology, hypoplasia of the internal carotid artery is exceedingly rare, occurring in less than 0.01% of cases, with underreporting potentially contributing to its rarity [8]. The condition is predominantly unilateral and more frequently observed on the left side. However, bilateral occurrences, though rarer, are possible. Given its scarcity, the condition is frequently detected incidentally during neuroimaging studies for unrelated neurological conditions. Comprehensive epidemiological data are limited, hampering precise prevalence determination [4].

## Conclusions

In this case report, the convergence of recurrent severe migraine headaches and the unexpected finding of left internal carotid artery hypoplasia sheds light on the intricate relationship between cerebrovascular anomalies and neurological symptoms. The rarity of hypoplastic internal carotid artery occurrences underscores the importance of vigilant clinical assessment and the necessity to consider anatomical variations in patients presenting with migraines. This case serves as a reminder that comprehensive evaluation, including detailed neuroimaging, is essential to unravel the potential underlying factors contributing to the clinical presentation. As medical understanding evolves, further research is imperative to elucidate the mechanistic nuances that link vascular anomalies and neurological disorders, ultimately guiding tailored therapeutic interventions for improved patient outcomes.
